# Introduced and invasive alien species of Antarctica and the Southern Ocean Islands

**DOI:** 10.1038/s41597-023-02113-2

**Published:** 2023-04-11

**Authors:** Rachel I. Leihy, Lou Peake, David A. Clarke, Steven L. Chown, Melodie A. McGeoch

**Affiliations:** 1 Securing Antarctica’s Environmental Future, School of Biological Sciences, Monash University, Victoria, 3800 Australia; 2 Arthur Rylah Institute for Environmental Research, Department of Energy, Environment, and Climate Action, Heidelberg, Victoria 3084 Australia; 3 Securing Antarctica’s Environmental Future, Department of Environment and Genetics, La Trobe University, Melbourne, Victoria 3086 Australia

**Keywords:** Invasive species, Biogeography

## Abstract

Open data on biological invasions are particularly critical in regions that are co-governed and/or where multiple independent parties have responsibility for preventing and controlling invasive alien species. The Antarctic is one such region where, in spite of multiple examples of invasion policy and management success, open, centralised data are not yet available. This dataset provides current and comprehensive information available on the identity, localities, establishment, eradication status, dates of introduction, habitat, and evidence of impact of known introduced and invasive alien species for the terrestrial and freshwater Antarctic and Southern Ocean region. It includes 3066 records for 1204 taxa and 36 individual localities. The evidence indicates that close to half of these species are not having an invasive impact, and that ~ 13% of records are of species considered locally invasive. The data are provided using current biodiversity and invasive alien species data and terminology standards. They provide a baseline for updating and maintaining the foundational knowledge needed to halt the rapidly growing risk of biological invasion in the region.

## Background & Summary

Collating data on alien species has seen much recent focus, including on integration of data types and sources, and on advances in the biodiversity informatics standards needed to support such data collation. These data now provide essential insight into the distribution, impact, and status of biological invasions worldwide (e.g.^1–3^). They are foundational in making evidence-based decisions for surveillance, early eradication, and control, and underpin monitoring and reporting on management success^4^. They also contribute to recent commitments made by the Parties to the Convention on Biological Diversity to continue to exchange information, use appropriate data standards, and regularly curate and maintain data on invasive alien species (CBD/COP/15/L.12). While there has been a step change in delivering country-level^2,5^ and taxon-specific inventories of alien species (e.g.^1,6^), some particular geographic and taxonomic data gaps still exist. One such gap is the Antarctic region. In part, this data deficit owes to the fact that the region south of latitude 60°S is managed as an international space through the Antarctic Treaty System^7^, thus largely excluding it from the application of international agreements of the United Nations, such as the Convention on Biological Diversity^8^. The Southern Ocean Islands, which encircle the continent, typically lie north of 60°S, and which form part of the Antarctic region^9–12^, are subject to several national jurisdictions.

From the earliest expeditions to Antarctica and the Southern Ocean Islands, humans have intentionally and accidentally introduced non-native species to the region^13,14^. On the continent itself, very few of these species have established self-sustaining populations away from human influence due to the severity of the climate, Antarctica’s isolation, and the early adoption of biosecurity practices by the Parties to the Antarctic Treaty^15^. Following the entry into force in 1998 of the Protocol on Environmental Protection to the Antarctic Treaty^16^, the introduction of domestic stock, sled dogs and non-sterile soil has been prohibited, and the introduction of living organisms subject to strict permit conditions.

In contrast, many alien species have established on the comparatively milder Southern Ocean Islands; some resulting in significant impacts on local biodiversity^17^. Notable examples include widespread cat, mice, and rat predation on endemic insects and seabirds^18–20^, vegetation damage caused by rabbits and other introduced mammals^21,22^, and the transformation of some plant communities by weeds^17,23^. These impacts have prompted the development of increasingly stringent biosecurity procedures across the region^24^, and invasive species control and eradication programs (e.g.^14,17,25–27^).

Despite the success of many of these practices in limiting the introduction, establishment and impact of alien species, biological invasions remain a key threat to Antarctic ecosystems. Recent research has shown that Antarctic ports are surprisingly well-connected to the global shipping network^28^ and physiological barriers to the establishment of alien species will likely weaken under future climate change scenarios^29–31^. Moreover, projections also indicate that the impacts of invasive alien species on their indigenous counterparts and on ecosystems are likely to grow into the future with increasing human occupancy of the region and with changing climates^32,33^. Given these expectations, the management of introduced and invasive alien species remains a priority for the region. For example, the Committee for Environmental Protection (CEP), which advises the Antarctic Treaty Consultative Parties (ATCPs) on management for the region, has made such species (non-native species in their terminology) one of its major priorities, reflected not only through its regular attention to the matter^34,35^, but also by the Non-Native Species Manual it has published and updates regularly^36^. Similarly, the management plans for most of the Southern Ocean Islands have a key focus on biosecurity and invasive alien species threat abatement (e.g.^37–39^).

Given these priorities, and the demonstrable value of consolidated, open and FAIR (Findable, Accessible, Interoperable, Reusable)^40^ inventories of biological introductions for both management and assessment of management actions, such an inventory should be a priority for the broader Antarctic region. Yet such an inventory, which complies with rigorous modern biodiversity informatics standards^41^, does not exist, despite multiple previous data collations^15,17,42^. One major consequence thereof is that, at present, apart from documentation of successful or unsuccessful eradication attempts, the ATCPs and the authorities responsible for the conservation management of the Southern Ocean Islands have no way of assessing the extent to which their, often stringent^24^, biosecurity procedures have had any effect.

Introduced and invasive alien species data for the region have previously been collated in various traditional forms. In 2005, Frenot and colleagues provided a narrative review of the current knowledge of alien species in Antarctica and the sub-Antarctic islands, including information on species distribution, impact, and origin^17^. Other contributions include regional checklists and presence-absence datasets for particular groups (mammals^14,21^; insects^43^; springtails^44^; spiders^45^; and mites^46^), systems (e.g., freshwater fauna^47^), and management areas (e.g.^15,39,42,48^). These resources differ in their geographic, temporal, and taxonomic scopes. For example, some datasets do not include more northerly islands, such as those of the Tristan da Cunha group and the Falkland Islands/Islas Malvinas, or the New Zealand sub-Antarctic islands, despite their historical biogeographic similarity to other islands in the region^9,10^. Likewise, information on species occurrence and evidence of impact (invasive status) are not consistently reported.

Here, we present what is, to our knowledge, the most complete and up-to-date record of introduced terrestrial and freshwater species in the Antarctic region. The dataset is formatted using existing biodiversity informatics standards and vocabularies developed by Darwin Core^49^ and the Global Register of Introduced and Invasive Species (GRIIS)^2^ to improve the interoperability of the records^50^. While the data fields and standards used are consistent with recent contributions^5^, this publication differs from other open data on introduced species in three critical ways: 1) it provides information at a level of governance appropriate for evidence-delivery in support of the Antarctic Treaty System and national governance of the Southern Ocean Islands; 2) it is neither country- nor specific taxon group-focussed; 3) in addition to fields variously present in existing sources (e.g.^17,51,52^), we provide population discovery dates (date of introduction, first observation and/or first published record) and the location-specific eradication status of taxa; 4) along with record level information sources. These additional data are essential for tracking the success of policy and management interventions for invasive alien species^53^, and contribute to improved knowledge and understanding of biological invasions in the region. The purpose of this dataset is thus to provide readily accessible, centralized baseline information on the occurrence, discovery, impact, and successful eradication efforts for introduced and invasive alien species across the Antarctic region.

## Methods

### Geographic coverage

**Fig. 1:**
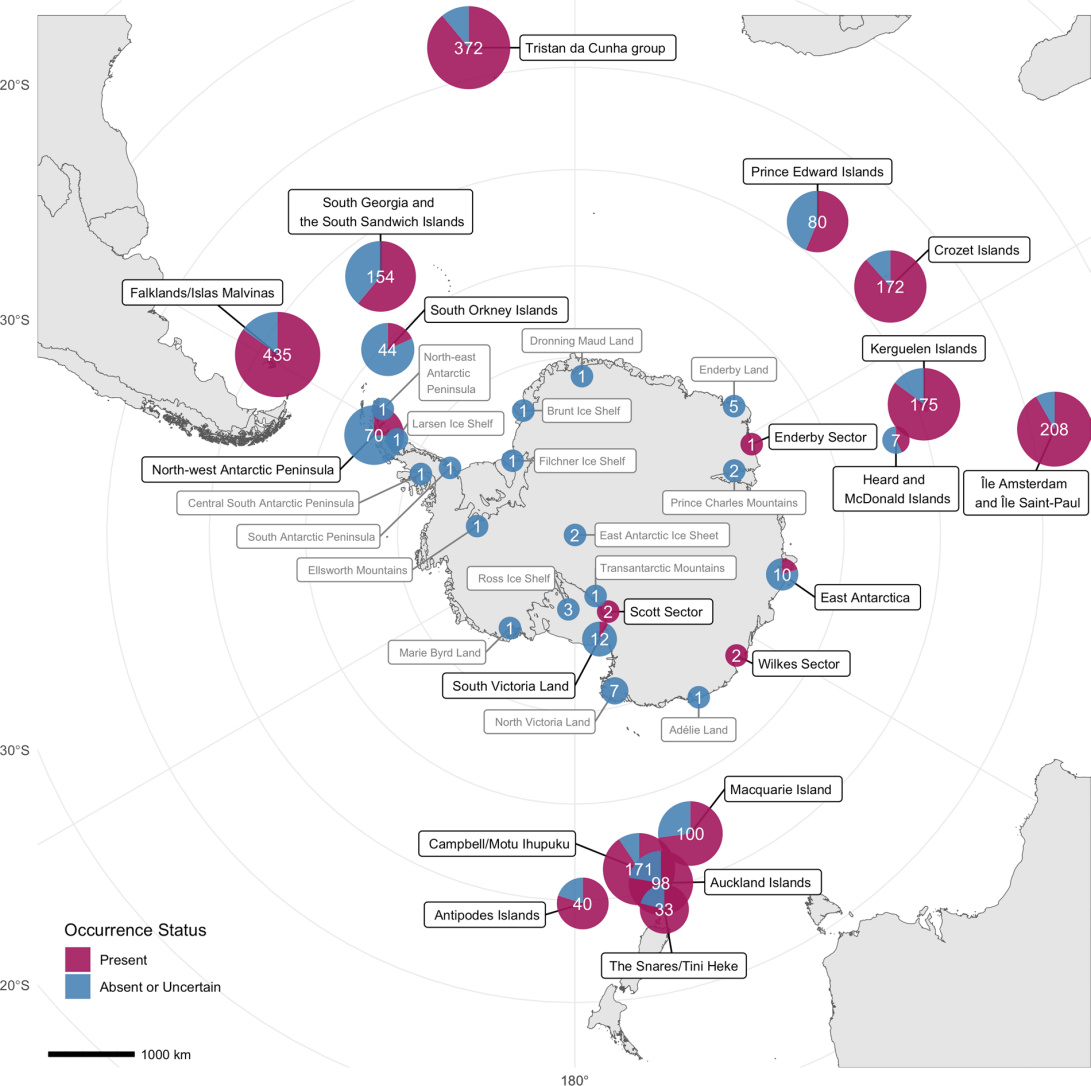
Spatial coverage of the data across the Antarctic. Number of alien species records across the Antarctic and Southern Ocean region. Values and point size indicate the total number of unique alien species or other taxon records per locality. Shaded points indicate the proportion of records currently listed as present (extant) at each locality (purple), versus the proportion of records with an uncertain or absent occurrence status in blue. Point sizes are log-scaled and constrained by a minimum size for localities with fewer than five records, for display. Localities without records with a present occurrence status in the dataset listed in grey text.

The dataset includes records from continental Antarctica, the maritime Antarctic islands, and the Southern Ocean Islands (Fig. 1). Here, in keeping with previous approaches (e.g.^10,12^), the Southern Ocean Islands include fifteen islands or archipelagos that straddle the Antarctic Polar Front or, if lying further to the north, are part of a biogeographically coherent grouping by virtue of phylogenetic relatedness of terrestrial species (i.e., the Falklands/Islas Malvinas, South Georgia and the South Sandwich Islands, Bouvetøya, Tristan da Cunha group (including Gough Island), Prince Edward Islands, Crozet Islands, Kerguelen Islands, Heard and McDonald Islands, Île Amsterdam and Île Saint-Paul, Macquarie Island, Auckland Islands, Campbell Island/Motu Ihupuku, Bounty Islands, The Snares/Tini Heke and the Antipodes Islands). The exclusion of the Chilean and Argentinian islands at the southern tip of South America (e.g., Tierra del Fuego and the Diego Ramírez Islands) recognises their closer association with the biodiversity of Patagonia, though noting that the biogeographic classification of islands across the broader Southern Ocean region remains fluid (e.g.^54^).

### Population of data fields

Records of 2390 extant and 676 extinct or status-uncertain species or other taxon introduced to Antarctica and the Southern Ocean Islands were collated from a systematic search of the scientific literature (between March 2021 and January 2023). The dataset contains an inventory of alien species records from terrestrial habitats and land-based waterbodies across sites, and information on their occurrence status, date of discovery, and whether there is evidence of an environmental impact at each locality. Lacustrine, intertidal, and synanthropic species only found within buildings were included, and entirely marine species were excluded from the dataset. Introduced taxon records from the earliest available date were included in the dataset, including historical records of alien species that have since failed to establish, species that have been eradicated or removed, and extant alien species (further detail below). Specifically, the data include 30 fields, describing information on taxonomic position, location, occurrence and eradication status, dates of first record or discovery, realised impact evidence and habitat information (Table 1).

**Table 1:** Fields in the dataset.

Field	Description
recordID	Unique record number
**Taxonomic information**	
acceptedNameUsage*†	The accepted name and authority of the taxon, according to GBIF
verbatimIdentification*	The name of the taxon as recorded during the data collection process
species	The accepted name of the taxon, given as a binomial name excluding subspecies and authorship information. The suffix “sp.” is appended for genus-level records
kingdom*/phylum*/class*/order*/family*	Higher level taxonomy, primarily according to GBIF
taxonRank*	The rank of the taxon. Terms: genus; species; subspecies; family; subfamily; order; variety; group
**Geographic coverage and location information**	
locationID*	Unique locality identifier, specific to the data set
higherGeographyID*	A statement about whether the locality is in Antarctica (south of 60°S), or on the sub-Antarctic and temperate Southern Ocean Islands (north of 60°S). Terms: Antarctica; sub-Antarctic
locality*	The biogeographic region or archipelago where the record occurs
locationRemarks*	A specific description of the place where the record occurs
countryCode_alpha2	The alpha-2 code of the country according to the ISO 3166 standard
countryCode_alpha3	The alpha-3 code of the country according to the ISO 3166 standard
**Establishment and eradication information**	
occurrenceStatus*	A statement about the presence or absence of the taxon at the locality. Terms: present; absent; uncertain
occurrenceRemarks*	Comments or notes about the occurrence
establishmentMeans*	Statement about whether an organism or organisms have been introduced to a given place through direct or indirect activity of modern humans. Cryptogenic species of unknown biogeographic origin are listed as ‘uncertain’. Vagrant records are natural occurrences of organisms outside their normal ranges and human-aided introductions where the degree of establishment is minimal. Terms: introduced; uncertain; vagrant
occurrenceReference	Citation/s for the verbatimIdentification, locationRemarks, occurrenceStatus, occurrenceRemarks and/or establishmentMeans data
eradicationStatus	A statement about whether the taxon was eradicated (or removed), or failed to establish or died out. Terms: eradicated; failed to establish or died out
eradicationYear	Year of eradication or local extinction of the taxon, if known
eradicationReference	Citation/s for eradicationStatus and eradicationYear fields
**Temporal coverage and dates on introduction**	
estimatedIntroductionDate	Date that the taxon was first introduced, or the estimated date for first introduction, written exactly as found in the source reference
firstObservationRecord	Date that the taxon was first observed (i.e., discovered), written exactly as found in the source reference
firstPublishedRecord	Year for the first published record
firstRecordReference	Citation/s for estimatedIntroductionDate, firstObservationRecord and/or firstPublishedRecord data
**Habitat and impact information**	
habitat*	The environment in which the taxon occurs or that it is associated with. Terms: freshwater; brackish; terrestrial; marine; host; and their combinations
isInvasive	A taxon is designated as invasive (‘yes’) in a locality using the systematic decision process outlined in Pagad *et al*. (2018)^2^, where ‘null’ indicates a taxon for which there is an absence of evidence of impact, and ‘no’ indicates evidence that the species population at that locality is not having an impact
invasiveReference	Citation/s for isInvasive designation

Accepted taxon names and higher taxonomic classifications were matched against the Global Biodiversity Information Facility (GBIF) Backbone Taxonomy^60^. An asterisk (*) indicates a Darwin Core term^49^, a dagger (†) indicates a term also used in the Global Register of Introduced and Invasive Species (GRIIS)^2^.

The data fields were populated from a systematic search of the scientific and grey literature, including peer-reviewed publications, existing species checklists, and government reports and other documents containing species lists, such as management plans^39^. For some Antarctic regions and taxa, alien species data have already been compiled (e.g.^14,17,42,43^). To ensure that search effort was given to each area of the Antarctic region, additional data were collected from a Web of Science search with Boolean strings, using combinations of regional place names and taxonomic groups (Table 2). Titles and abstracts were screened for their relevance to the dataset. Species information on the occurrence status, invasive status, date of first discovery, and eradication status and date (where applicable), were extracted from the literature. The reference lists of relevant publications were also screened for additional sources. Lastly, key publications for the species occurrence data (i.e., references that listed ≥ 10% of the occurrence records per broad taxonomic group) were back-searched in Google Scholar for resources that cited these documents to capture recent additions to the literature.

**Table 2:** List of Boolean string terms used to systematically search the Web of Science database for scientific literature relevant to the Antarctic alien species database.

Regions	Taxonomic groups
(Amsterdam Island* OR Île Amsterdam OR Nouvelle Amsterdam)	(Acari* OR mite*)
(Amsterdam) AND (St. Paul OR Saint Paul)	(Annelid* OR worm*)
(Antarctic* OR Antarctiq* OR Antartid*)	(Aves OR bird*)
(Antipodes Islan*)	(Bryophyt* OR moss* OR liverwort*)
(Apôtres* OR Apostle Isl*) AND (Crozet)	(Collembola* OR springtail*)
(Auckland Islan*)	(Crustacea* OR slater* or woodl* NOT marine)
(Bouvet Island* OR Bouvetøya)	(fish* OR salmon* OR trout*)
(Campbell Island* OR Motu Ihupuku)	(flora OR vegetation OR plant*)
(Cochon* OR Pig Island*) AND (Crozet)	(fung* OR mould* OR mushroom*)
(Crozet Island* OR Îles Crozet)	(insect*)
(East Falkland*)	(mammal* OR livestock*)
(Est* OR East Island*) AND (Crozet)	(Mollusca* OR snail* OR slug*)
(Falkland* OR Islas Malvinas)	(Myriapod* OR millipede* OR centipede*)
(Gough Island*)	(Platyhelminth* OR flatworm*)
(Grand Terre) AND (Kerguelen)	(spider* OR arachnid*)
(Heard Island*)	
(Heard) AND (McDonald)	
(Inaccessible Island*)	
(Kerguelen)	
(Macquarie Island*)	
(Marion Island*)	
(McDonald Island*)	
(Nightingale Island*)	
(Pingouins* OR Penguin Island*) AND (Crozet)	
(Possession*) AND (Crozet)	
(Prince Edward Island*)	
(Saint Paul Island* OR St. Paul Island* OR Île Saint-Paul OR Île Saint Paul)	
(Snares Island* OR Tini Heke OR The Snares)	
(South Georgia Island*)	
(South Orkney Island*)	
(South Sandwich Island*)	
(sub-Antarct* OR Southern Ocean Island* OR subantarct*)	
(Tristan da Cunha OR Tristan group OR Tristan Island*)	
(West Falkland*)	
(South Shetland Island*)	

In total, 525 searches were conducted, using all combinations of key regional location and broad taxonomic group terms. Terms to refine searches to introduced species (“(introduced OR alien* OR invasive* OR non-native*) AND (species)”) were also included.

Information sources used to populate the data fields are recorded in four fields in the dataset (Table 1). Sources used to populate the species name (verbatim), taxon rank, locality, establishment means, and/or occurrence status fields were recorded in the ‘occurrenceReference’ field. Sources used to populate the estimated introduction date, date of first observation, and/or the date of the first published record are recorded in the ‘firstRecordReference’ field. References used to designate the invasive status of a given taxon in a locality were recorded in the ‘invasiveReference’ field, and the eradication status and year sources were recorded in the ‘eradicationReference’ field. Values in these reference fields correspond to the full list of resources used to compile the dataset, provided in the “AntarcticAliens_references.csv” file (n = 384)^55^.

### Taxonomic information

#### Coverage and inclusion criteria

Alien records from all terrestrial and freshwater taxonomic groups in the Antarctic region were considered in data collection. The most well-known and successful groups in the region include vascular plants, mammals, insects, birds, spiders, annelids, and springtails^14,17^. Alien species intercepted in transport pathways to the Antarctic region (e.g.^56,57^), were not considered. There is limited evidence on the occurrence and origin of alien marine species and microorganisms in Antarctica^58,59^. These groups are therefore not included.

Records were included if they referred to:

1. a taxon that had been introduced (either intentionally or accidentally) into an Antarctic area outside of its native range, including synanthropic species that are only associated with sites influenced by humans (e.g., within research stations in Antarctica),
2. a taxon that has spread to a new area beyond its native range from an adjacent introduced population (secondary introductions), including native Antarctic species that have been introduced to new areas within the region by humans, or,
3. cryptogenic species of unknown biogeographic origin, in other words species that cannot be ascribed as native or introduced at a particular Antarctic locality.

Here, the terms ‘alien’, ‘introduced’, ‘adventive’, ‘non-indigenous’, ‘non-native’ and ‘exotic’ are considered synonymous, and describe all taxa introduced to places outside their native ranges, including vagrants, domestic species, established or naturalised aliens, invasive alien species, and species that were introduced to areas but have since become locally extinct because they failed to establish a self-sustaining population, died out following establishment, or were eradicated.

#### Harmonisation and normalisation

Species names (or other taxon-level information) are recorded verbatim from the literature in the ‘verbatimIdentification’ field. Taxa are recorded at the most specific taxonomic level (typically species level) provided in the literature (see the ‘taxonRank’ field). For records identified to a genus-level taxonomic rank, the ‘sp.’ suffix was appended to the genus name in the ‘verbatimIdentification’ field.

The Global Biodiversity Information Facility (GBIF) Backbone Taxonomy^60^ was used to harmonise the taxonomy of records included in the dataset in a reproducible and standardised way. Higher taxonomic classifications (kingdom, phylum, class, order, family) and the currently accepted taxon names and authorities (‘acceptedNameUsage’ field) were matched with the verbatim taxon names extracted from the literature, using the ‘get_gbif_taxonomy’ function in ‘traitdataform’ package (v. 0.6.8)^61^ in R^62^. Synonyms and misspelled names were resolved in this process. Over 85% of names in the dataset were matched at 95% or greater confidence (Table 3). Names that were not exactly matched were checked and modified where appropriate. In some cases, these taxa were manually harmonised using the GBIF taxonomy by providing additional information on their authority or higher taxonomy from the literature to the ‘get_gbif_taxonomy’ function. For 26 taxa, it was not possible to harmonise their higher taxonomy using this approach, and their classifications are given as ‘NA’ in the dataset (Table 3).

**Table 3:** Taxonomic matching results using the Global Biodiversity Information Facility (GBIF) Backbone Taxonomy^60^.

Match type	Kingdom	No. names	Percentage in taxon
High confidence (≥95%)	Plantae	594	91.24
	Animalia	406	79.76
	Fungi	41	93.18
Moderate confidence (80–94%)	Plantae	44	6.76
	Animalia	86	16.9
	Fungi	3	6.82
Low confidence (<80%)	Plantae	1	0.15
	Animalia	2	0.39
	Fungi	0	0
Manually harmonised	Plantae	12	1.84
	Animalia	15	2.95
	Fungi	0	0
**Total**		**1204**	

Results show the number and percentage of unique names in the dataset, per kingdom, with higher taxonomic classifications standardised with high, moderate, and low confidence using the ‘get_gbif_taxonomy’ function in the ‘traitdataform’ R package^61^. Some names were manually matched using the GBIF taxonomy by providing additional information on their authority or higher taxonomy to the function.

The accepted name of the taxon, given as a binomial name excluding subspecies and authorship information, is also provided in the ‘species’ field. The suffix ‘sp.’ is appended for genus-level records. For 18 invertebrate taxa, including the well-known alien aphid, *Aphis gossypii*, the ‘get_gbif_taxonomy’ function did not harmonise the record to species level. In these cases, the ‘species’ information was matched with ‘verbatimIdentification’.

### Location information

Location details were standardised and translated during data collection. A field (‘locationID’) was added to match the locality descriptor, and verbatimLocality to provide further detail about the place associated with the occurrence record. Furthermore, higherGeographyID was included to indicate whether localities sit within the Antarctic Treaty Area (i.e., south of 60°S), where the management of non-native species threats is the responsibility of the Consultative Parties to the Antarctic Treaty^16,63^. North of 60°S, the management of the Southern Ocean Islands falls under the national jurisdictions of the countries that have annexed each island or archipelago^24^. The ISO 3116-1 Alpha-2 and Alpha-3 country codes are included in the dataset to identify these various governance arrangements and to facilitate dataset integration with other lists^2^.

### Establishment and eradication information

Statements about the occurrence status and establishment means were derived from the literature. All alien taxa were considered introduced unless a source stated that the species has an unknown biogeographic origin and could not be designated as native or introduced to a given locality (i.e., an uncertain establishment means or cryptogenic record), or that the species is a vagrant, casual, transient, non-resident or ephemeral species at a locality (vagrant establishment means)^41^. Likewise, records of alien species were denoted as ‘present’ (occurrenceStatus), unless sources stated that they are absent from localities, or that their occurrence is uncertain or doubtful.

Information on the eradication status and date of eradication were also extracted from the literature (fields: eradicationStatus, eradicationYear, eradicationReference). Species that are now absent from the area of introduction were categorised as ‘eradicated’ or ‘failed to establish or died out’, depending on the process that caused their extirpation (eradicationStatus). Eradicated records include taxa that have been actively and successfully eradicated or relocated from Antarctic localities. Species that failed to form self-sustaining populations or completely died out after establishing were categorised as ‘failed to establish or died out’. Eradication year is the date the population was determined to be absent from the locality, where available.

### Temporal coverage and dates of introduction

Three types of dates were included in the dataset to capture the different ways in which alien species introductions are reported (estimatedIntroductionDate, firstObservationRecord, firstPublishedRecord). Estimated introduction date is the date the taxon was first introduced or thought to have been introduced to a locality, stated explicitly as such in the literature. For records of aliens discovered after they had established, reproduced, or spread, the date provides a ‘first record’ and is assumed to post-date the introduction date^64^. Dates are recorded in a variety of formats as found in the literature (e.g., “1940s”, “before 1962”, “early nineteenth century”). First observation record is the date that the taxon was first observed *in situ*, recorded as a year or range of years (with the meaning consistent with that of ‘first record’, *sensu*^65^). First published record is the year of the publication first documenting the introduction. There is often a publication lag between when species are first observed, and the record is published.

### Habitat and impact information

Species-specific habitat information was extracted from GRIIS^5^ to facilitate research requiring data integration across sources. This high-level environmental information is equally relevant for the Antarctic context. For taxa not included in the GRIIS dataset, habitat information was sourced from the literature.

The status of an alien species occurrence presence at a locality was designated as invasive following the method used by the Global Register of Introduced and Invasive Species (GRIIS)^2,5^ and summarised here. Species present at a locality were designated as invasive if any authoritative source described an environmental impact, and/or determined that the taxon is widespread, spreading rapidly, or present in high abundance. Species were also designated as invasive in cases where any authoritative source determined they were invasive at that locality. Records are thus assigned the value ‘yes’ if there is evidence of an invasive impact, ‘no’ if there is evidence that the record has a non-invasive status, and ‘null’ if there is an absence of evidence about the impact, in the ‘isInvasive’ field. Evidence of a non-invasive status may include statements such as ‘synanthropic’, ‘vagrant’, ‘occasional’, ‘few individuals’, ‘rare’, ‘minor impact’, and ‘restricted distribution’. This evidence is based on their reported extent or impact and should be revised when formal impact assessments (e.g.^66^), are completed (see Usage Notes). Where available, a statement on the occurrence and/or invasive status of species is included in the ‘occurrenceRemarks’ field. For eradicated taxon, the ‘isInvasive’ data refers to their pre-eradication status.

Standards for what constitutes an invasive species impact, widespread distribution, high abundance, and/or rapid spread do not exist in the ecological literature (although high level classification systems do^66^) and are context- and taxon-dependent^67^. Species impacts may also change over time, for example, as local population sizes fluctuate. The ‘isInvasive’ status of the records in this dataset therefore reflects information available at the time of publication of the associated impact reference and the content of these publications interpreted as outlined above.

### Data summary

There are currently 3066 records of 1204 introduced or cryptogenic taxa in the Antarctic and Southern Ocean Islands dataset. Most records were identified to species (92.5%) or genus (4.2%) level, with the remaining records representing subspecies (2.1%), families (0.6%), varieties (0.4%), subfamilies (<0.1%), groups (<0.1%) or orders (0.1%).

Most records and species are from the Southern Ocean Islands (records: 92.9%, n = 2848; species: 93.4%, n = 1125), with only 7.1% (n = 218) of records and 135 species in the Antarctic Treaty area (Fig. 1). Localities with permanent human settlements, including the Falklands/Islas Malvinas and Tristan da Cunha, have the most non-indigenous species records in the region.

**Fig. 2:**
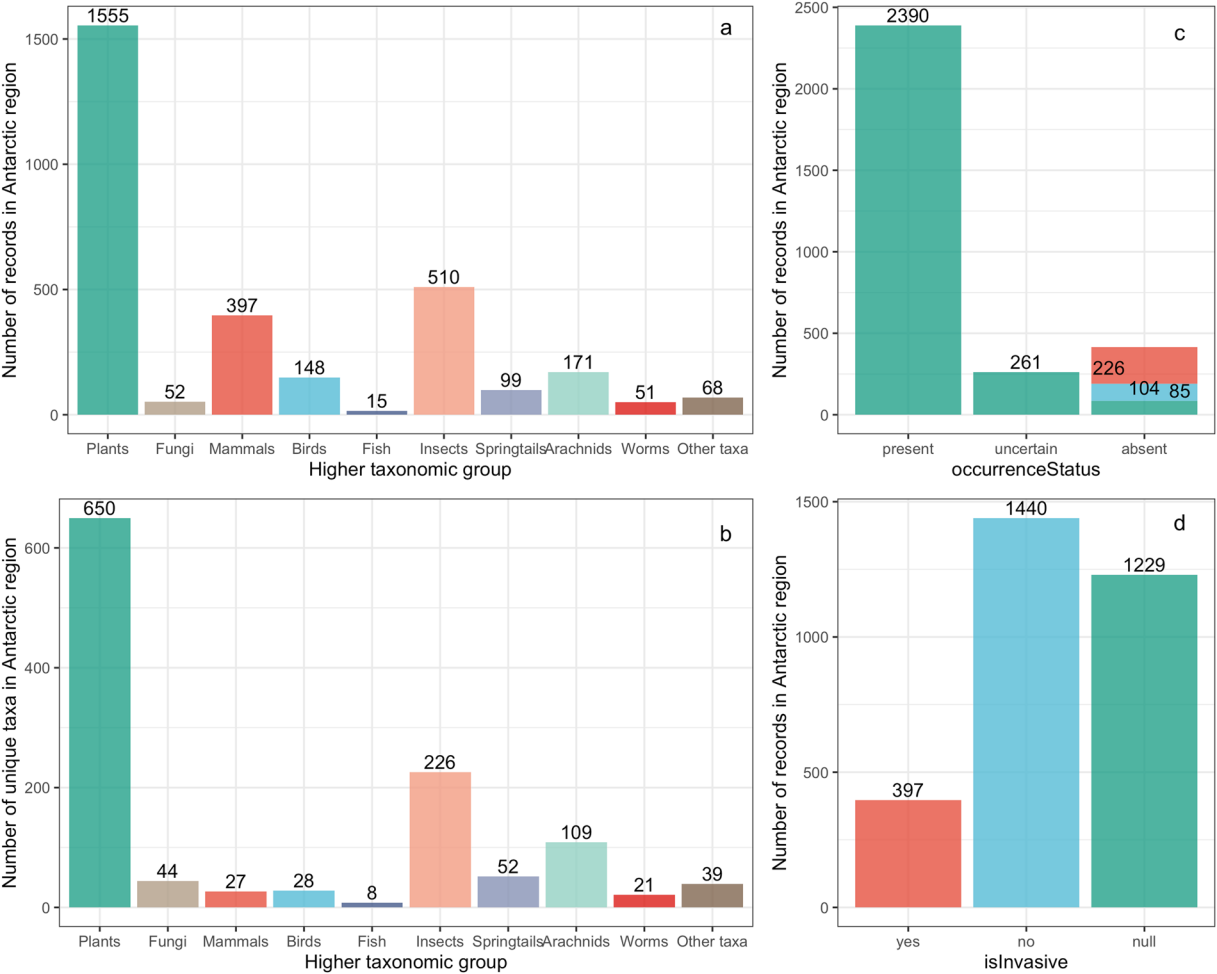
Data records summary. (**a**) Number of records and (**b**) unique taxa per major taxonomic group introduced to the Antarctic and Southern Ocean islands. (**c**) Number of records with a present, uncertain, or absent occurrence status in the Antarctic region. Absent records are partitioned by their eradication status: eradicated (red), failed to establish or died out (blue), or unknown eradication status (green). (**d**) Number of records per isInvasive status, where “yes” indicates an invasive species, “no” indicates a non-invasive species (e.g., transient, synanthropic, restricted distribution), and “null” indicates an absence of information (i.e., population-level invasion status). Values above bars indicate the number of records or taxa in each group.

Vascular plants and insects make up the majority of alien species records and unique taxonomic records across the Antarctic region (Fig. 2a,b). Approximately 78% of records refer to extant alien species (occurrenceStatus “present”), with the remaining records representing uncertain records or species that have been eradicated, failed to establish, or died out (Fig. 2c). For establishment means, most records are introduced (94.8%, 2908 records), with only 3.5% of records as vagrant (n = 107) and 1.7% of unknown biogeographic origin (i.e., a cryptogenic or uncertain establishment means, n = 51).

At a population level, 12.9% of populations (locality by species records) are considered invasive, 47% of records have non-invasive status (e.g., transient, synanthropic; see occurrenceRemarks), and, for 40.1% of records, there is an absence of evidence of impact in the dataset (Fig. 2d). At a species level, 14.9% of taxa (n = 180) are considered invasive at one or more Antarctic and Southern Ocean Island localities, 54.5% of species have a non-invasive status (n = 656), and for 30.6% of species, there is an absence of evidence of impact (n = 368).

## Data Records

The Antarctic and Southern Ocean Islands alien species data are stored in the Figshare data repository^55^. The main data table is contained in a single comma-separated file (.csv format), entitled ‘AntarcticAliens_dataset.csv’. Each row below the header represents a record of a specific alien species (or other taxon) introduced to a specific location (locality) (n = 3066). Columns contain information on the taxonomic position of species, occurrence status, date of first introduction, habitat, invasive status, and eradication status (if applicable) (n = 30). A list of column names, descriptions and field terms is provided in Table 1. Field names are consistent with Darwin Core^49^ and GRIIS terms^2^, where possible, to allow data interoperability^50^. Additional, non-standard terms describing introduction dates and eradication status provide information on the history of alien species records in the region (Table 1).

The full list of the references referred to in the main data table and their Digital Object Identifiers (DOIs), International Standard Book Numbers (ISBNs), or Uniform Resource Locators (URLs), where applicable, are contained in the single comma-separated file (.csv format), entitled ‘AntarcticAliens_references.csv’. Reference numbers (‘referenceNo’) match to the reference numbers entered in square brackets in the four reference fields in the main dataset (‘occurrenceReference’, ‘firstRecordReference’, ‘invasiveReference’, ‘eradicationReference’). Brackets were used to separate reference numbers, to improve the searchability of these records.

## Technical Validation

Where available, multiple independent sources were used to populate the dataset for each record and were cited in the four reference fields (Table 1). Some occurrence records from existing Antarctic datasets (e.g.^43^), that could not be verified by an independent source were excluded. Taxonomic and habitat data were harmonised using the process described above (see Methods), to standardise data collection and remove duplicated occurrence records from the dataset. During this process, necessary changes were made to correct spelling errors or inconsistencies, consolidate records of synonyms, and standardise the higher taxonomy information. Accepted and verbatim name fields are provided to facilitate ongoing validation and data integration.

## Usage Notes

### Completeness

**Fig. 3:**
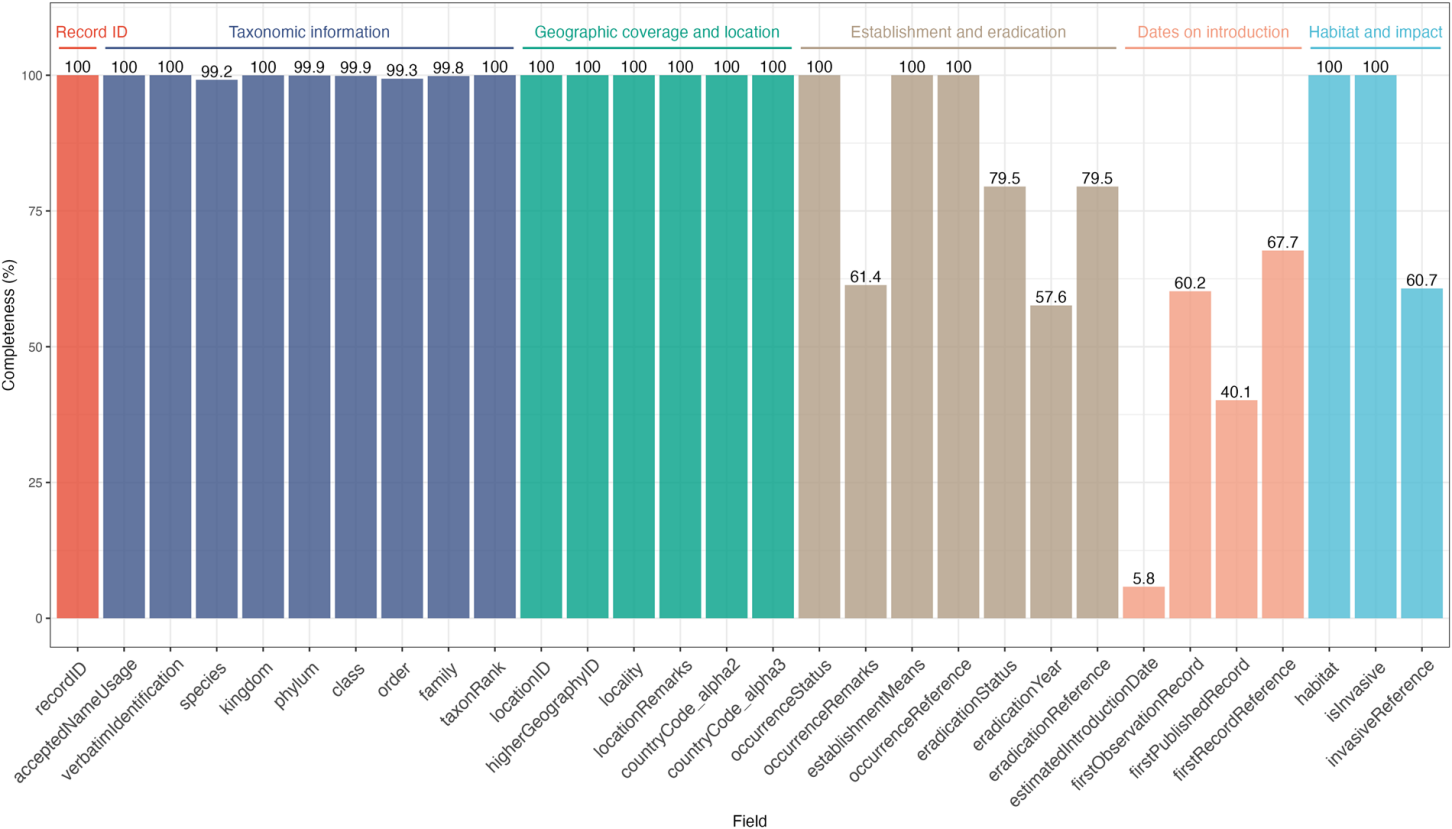
Dataset completeness. Percentage of records with data available for each field in the Antarctic and Southern Ocean Islands alien species dataset. Values above the bars indicate the percentage completeness for each field. For the eradication status, year and reference fields, completeness is the percentage of records with an ‘absent’ occurrence status with data available in these fields.

The dataset was populated from a systematic literature search between March 2021 and January 2023. In total, 384 sources were used to identify 3066 records of alien species in the region. Fields describing the taxonomy, location, and occurrence status of these records are mostly complete (99.2–100% complete; Fig. 3). Information on the discovery dates (5.8–60.2% complete records for estimatedIntroductionDate, firstObservationRecord and firstPublishedRecord fields), invasive status (60.7%) and eradication status (79.5%) and year (57.6%) are less complete in the current version of the dataset and will be the focus of ongoing dataset revisions. These gaps may be due, in part, to a lack of information about these fields in the literature, particularly for the date of accidental or secondary introduction events, or the date when alien populations died out in the area of introduction. In addition, primary reasons for records of alien species potentially not captured in the literature search include: 1) a time lag between when species are first observed or eradicated and when these events are documented in the literature, 2) language barriers to data collection using a scientific literature review method, 3) the misclassification of taxa in particular sites as native, 4) literature that is not widely cross-referenced, or, 5) under-reporting of introductions or translocations at stations associated with food or cargo imports^68^, including synanthropic aliens.

The location of most Southern Ocean Island records in the dataset is recorded at an island scale (‘locationRemarks’, e.g., East Falkland Island). For some records, it was not possible to identify which island or islands in an archipelago or region were relevant, and their locations are only provided at regional scale (‘locality’, e.g., Falklands/Islas Malvinas). Additional information on these alien species distributions could be added to the dataset to increase the spatial resolution of these records. The size of the localities in the dataset, and therefore the spatial resolution of the records, varies considerably across the region (e.g., Grand Terre Island >6500 km^2^ vs. Hallett Station <1 km^2^). Similarly, dogs (*Canis familiaris*) were extensively introduced to Antarctica between 1899 and 1994^14^. The number of these records for the continent (area south of 60°S) in the current version of the dataset under-represents the spatial extent of these introductions.

### Uncertainties and operability

This dataset is intended to be an updateable, versioned repository for information on the occurrence and status of alien species in the Antarctic region. Common to all alien species lists^69^, there are knowledge gaps and uncertainties in the Antarctic and Southern Ocean Islands alien species dataset. Alien species invasions are spatially and temporally dynamic processes that require regular surveillance and surveys to maintain current species lists and monitor the establishment, spread, and impact of aliens. Alien species checklists become out-of-date as soon as new species are introduced or detected, or the known distribution or environmental impact of extant alien species changes (whether due to their success or failure, or additional research)^5^. Taxonomic revisions and the re-examination of records may also update alien species identities, change higher taxonomic classifications, or correct misidentified specimens or incorrectly synonymised records.

This spatially and temporally specific dataset can be used to: 1) assess the status and changes in the introduction and invasion across the region, 2) identify gaps in taxonomic, distribution, or invasive status data to prioritise research and surveillance efforts, 3) assess the coverage of eradication programs, 4) identify invasion risks from neighbouring localities across the region, and 5) provide evidence to support policy decisions and reporting on biodiversity threats and management actions.

If the end-user intends to assess spatial or taxonomic patterns in Antarctic introductions, they should be aware of possible sources of bias in the dataset. First, in spatially explicit biodiversity datasets, there is often bias in the localities of records due to greater research or survey efforts in certain places, depending on factors such as accessibility, level of management, research priorities, availability of biological expertise, or places or species of interest or impact^69^. Bias also occurs where the level of research interest or taxonomic expertise differs across groups (e.g., records of Fungi in the dataset are predominantly from the Falklands/Islas Malvinas; whereas the most recent systematic reviews of spiders and mites across the region were published several decades ago^45,46^).

The purpose and extent of historical and contemporary human activity varies substantially across the Antarctic region. Several Southern Ocean Islands, including the Falkland Islands/Islas Malvinas and Tristan da Cunha, have permanent human settlements, agriculture, and industry. Other islands have historically been occupied by pastoral settlements (e.g., Île Amsterdam, Kerguelen Islands, Auckland Islands, Campbell Island) and onshore sealing and whaling operations (e.g., South Georgia, Marion Island, Kerguelen Islands, Macquarie Island, and the South Shetland Islands)^14^. In these places, alien propagule pressure may be high due to their connectivity with global trade and travel networks. Furthermore, some alien introductions, such as ornamental plants, pets, and species of agricultural importance (e.g., forestry trials), may not be well documented on alien checklists because of their perceived social or economic value at the time of introduction.

In contrast, access to some Southern Ocean Islands (e.g., Prince Edward Island, Heard and McDonald Islands, Judge and Clerk Islands, and Bishop and Clerk Islets) is highly restricted. Some Antarctic island localities are simply inaccessible (e.g., Bouvetøya, Scott Island, Balleny Islands). These islands may experience a lower alien propagule pressure; however, biological surveys are also less frequent, and some introductions may not have been detected. Lastly, due to the various governance arrangements for Antarctica and the Southern Ocean Islands, policies and practices on biosecurity, and alien species monitoring and control, vary across the region^24^. These differences mean that some Antarctic localities, particularly on the continent, are more likely to have complete information on alien introductions than other regions, creating a potential geographic bias in the completeness of the dataset that needs to be considered when conclusions are drawn about spatial patterns of invasion from this dataset.

The field ‘occurrenceStatus’ should always be read in conjunction with the field ‘eradicationStatus’ for any assessments of current levels of invasion at localities in the region. The dataset includes information on introduction dates in three formats: estimated introduction date, year of first observation, and first published record (Table 1). While records in these fields are incomplete (data available for 5.8–60.2% records), 67.7% of records have at least one introduction date available, and 35.6% of records have two or more dates available.

The existence of particular taxonomic and geographic gaps in invasive alien species knowledge are widely appreciated, both for the Antarctic and elsewhere^69^, and a subject of the current assessment of invasive alien species by the Intergovernmental Science-Policy Platform on Biodiversity and Ecosystem Services (https://www.ipbes.net/invasive-alien-species-assessment). Ongoing collaborative and coordinated efforts amongst nations and researchers will be necessary to address them. With the publication of these data, a number of avenues for communicating and updating the information become possible, including through the Antarctic Biodiversity Portal (biodiversity.aq), and linking it with the Global Register of Introduced and Invasive Species^2,5^.

## Data Availability

No custom code was used to generate or process the data presented in this manuscript.
